# On Tumours of the Uterus and Its Appendages

**Published:** 1847-04-01

**Authors:** 


**On Tumours of the Uterus and its Appendages.** (Jacksonian Prize Dissertation.) By *Thomas Stafford Lee,* M.R.C.S. E., &c. London: John Churchill, 1847.

Mr. Lee was induced to publish this Essay from its having been selected for the Jacksonian Prize. The author appears to have been a diligent student of obstetric medicine, as he tells us that he gained Dr. Murphy's gold medal at the London University some years since, and that he has availed himself of opportunities, both in Paris and London, for becoming practically acquainted with diseases of the female sexual organs. In estimating the merit of the work, it is necessary to bear in mind that the author is still young—that his experience on the diseases he writes upon is necessarily limited—and that he has had to collect materials principally from other resources, and to arrange, record, and compile them in an exact and faithful manner. The class of diseases which the Essay comprises is the least accessible, for practical investigation, to the student or general practitioner, of any which come before him; and novel views, or new facts of importance, are not to be expected, excepting where the field for observation is extensive. Mr. Lee has had the wisdom not to attempt too much. He appears to have assigned himself a very useful and important task; and, without straining after the display which so often dazzles the young author of being an original observer, he has investigated with much diligence the preparations in some of the best museums in London, and has taken great pains to collect authentic information on the subject of his dissertation. Hence it is that the body of the work is good, and one or two subjects, particularly that on the diseases of the ovary, is entitled to a still higher commendation. The chapter on Ovarian Dropsy is the fullest and the best we have yet met with; and, with the interest which at present attaches to cystic diseases of the ovary, it is one which well supplies a want which was generally felt. But, while it is our pleasing duty thus favourably to notice the most important part of Mr. Lee's Dissertation, we feel it equally incumbent on us to reprehend his style of composition. It is not only slovenly but faulty, and sometimes ungrammatical. There are numerous errors which even a superficial revision ought to have corrected; and we have grievously to complain of the perplexing way in which the personal pronouns I, we, and you, are used. We do not wish to attach too much importance to mere style, and we are far from being over-fastidious about it. We would carefully discriminate between the substantial merits of a work like Mr. Lee's, and the defect we have hinted at. But a clear perspicuous composition—an easy expression of thought or narration of facts—ought to be the first attainment, an educational rudiment, of every member of the medical profession.

The harmful and comparatively harmless qualities of the tumours which attack the uterus form the main division of this part of the Essay. The latter or benign tumours are first considered—and subsequently the more malignant growths. The five chapters which include the subdivisions under these two primary heads, refer respectively to Fibrous Tumours—Polypoid Tumours—Soft Polypi—Cauliflower Excrescence of the Os Uteri—and Encephaloid Polypi.

Mr. Lee ranges the fibrous tumours, as other authors have done, according to their situation. Some spring from the sub-peritoneal tissue and grow outwards, carrying the serous membrane before them, which first partially and then completely covers them, and forms a moveable stalk connecting them to the womb. Others, again, are imbedded in the proper structure of the uterus, which forms a loose investment around them, from which they may be enucleated: and, lastly, they may grow inwards towards the cavity of the womb—the lining membrane of which becomes their superficial covering. Our author says that the latter variety is the most frequent; in proof of which, he has tabulated 74 museum preparations—from which it appears, that 19 distend the cavity, 18 are attached to the anterior wall, 18 to the fundus externally, 4 only to the anterior wall, 4 to the posterior part of the cervix, and 5 in all parts of the womb. This table is interesting, as shewing what we believe a far more extended numerical investigation would prove correct; namely, the comparative immunity from these growths, which the front wall of the body and the neck of the womb enjoy. But we doubt the other alleged fact—of the greater frequency of the submucous variety over the central and subserous situations. If Bayle be right in declaring that the fifth part of women, above the age of 35 years, are affected with fibrous tumours—and Dr. Lee does not think he has over-estimated their frequency—we need scarcely say that such a meagre table as 74 museum preparations are just as likely to prove what is false as what is true. The fact is, in the large hospitals, there is a wholesale annual waste of these tumours, and those only are preserved which exhibit some peculiarity or special mark of interest. And hence we believe that Mr. Lee's table has been constructed from picked cases, to which the hundreds which have been destroyed ought to have been added before any undeniable inference could be made out. So far as our own experience goes, the submucous variety is happily the most rare, and we say this not only from our general impression from post-mortem examinations, but also from the practical fact that fibrous growths which cause bleeding from the uterus (the symptom which mostly attends the submucous tumours) are not very frequent. Our author has described very fully the morbid anatomy of these growths, and the changes they undergo. A microscopic analysis of them, from various situations in the uterus, "invariably presents a cellulo-fibrous appearance." Their tendency to pass into a cartilaginous or bony state, and the way in which the calcareous deposit sometimes forms a superficial layer, enclosing or shelling a growth, is noticed, and illustrative preparations referred to. The vascularity of fibrous tumours has been denied, but Mr. Lee correctly, we think, believes them to have a supply of arteries, the veins being collected around them.

"At no distant period was it affirmed that fibrous tumours were not vascular, and that they could not be injected: and this opinion was confirmed by the fact, that they remained uninjected, while all other parts of the womb had become quite red with injection. A preparation of this sort is preserved in the Museum of St. Bartholomew's Hospital, No. 10, Series 26. But, however, this art of injecting, during the last few years, has been better understood, and structures before considered non-vascular have been completely injected: and this is the case with fibrous tumours of the uterus. In the Museum of Guy's Hospital there are three preparations illustrating this fact, $2268^{86}$, 2270, and $2266^{32}$; in all these cases the injection has penetrated the morbid mass.

"Although uterine tumours can be injected, the vessels which are distributed to them are very small and few in number; and some tumours (even in the same uterus) cannot be injected at all. The vessels which penetrate the tumour are given off from the cellular cyst around them, which is extremely vascular: and it is from this layer that those fearful hæmorrhages arise when the tumour protrudes into the cavity of the uterus. We are thus supplied with the reason why these bodies do not undergo the changes consequent on inflammation, but only those of disorganization; for, if inflammatory action is set up in the cyst and the surrounding tissues of the womb, the small vessels passing to the tumour become obliterated, the supply of blood to the tumour is cut off, and the tumour itself dies.

"No veins are observed in the structure of these tumours; they only appear to be collected on their surface, where they are large and varicose: Savrard states that they are sometimes as large as the crural veins. They have been carefully injected, but no injection passes into the tumour." P. 9.

The general symptoms which accompany fibrous tumours of the uterus are common to other affections, and a vaginal examination, with the assistance of the uterine sound, afford the principal means of diagnosis. Mr. Lee extols the use of the sound in the investigation of uterine tumours, and he expresses an opinion that, when "the profession becomes more acquainted with the instrument, the diagnosis of abdominal tumours will be more correct." There are several affections with which fibrous tumours of the uterus may be confounded, and Mr. Lee marks the means of diagnosis in inflammatory indurations of the uterus, pregnancy, ovarian dropsy, other abdominal tumours not connected with the uterus, and polypi within the cavity of the womb. Of these, the most important in a practical point of view are the circumscribed indurations which follow inflammatory attacks, or even congestions of the womb, and which closely simulate fibrous growths. M. Lisfranc has directed attention to this subject, and it is well worthy careful consideration. This author notices the great difficulty in distinguishing between the two, and he mentions the fact, that in his earlier practice he perpetually regarded these indurations as fibrous growths. Mr. Lee describes the diagnostic marks of these affections in the following manner:—

"The difficulty of diagnosis between these two diseases is so great, that it led M. Lisfranc to believe that they could not be distinguished, except when the fibrous tumour became polypoid. But in inflammatory action of the uterus the constitutional fever appears at the commencement of the disease, almost before the swelling; while in fibrous tumour the mass is distinctly felt before the constitutional symptoms arise: there is also great pain on pressure. In hard tumours the swelling is more defined, and they are not usually painful on pressure. But the most diagnostic mark between these diseases is the effect of treatment on them. If the one yields to treatment, it may then be considered to depend on induration; as fibrous tumour has, according to some, never been reduced." P. 19.

In the diagnosis between pregnancy and fibrous growths, Mr. Lee depends a great deal on the areolar changes. He says that, in cases of fibrous tumour of the uterus when the disease has caused sufficient irritation as to induce the patient to apply for advice, he has found "that the breasts have, in a great majority of cases, been enlarged and tumid, that the areola has, in eight cases out of ten, been enlarged and darkened: that, in the same number, the follicles have been more or less numerous, in some they have been remarkably distinct: but that in only one case out of ten (and that a very suspicious case) was there any moisture or œdema of the nipple or areola present." Besides, the fresh, raised, varnished look of the areola (for we do not think the word œdema describes the change fairly), the character of the swelling of the gravid womb, its even surface, regular development, elastic and doughy feel, forms an appreciable difference between pregnancy and large fibrous growths. "In disease," says our author, "the tumour is irregular and of a stony hardness, no placental murmur or fœtal heart is heard." Undoubtedly the main sign of pregnancy, which outweighs all others in value and easy appreciation, is the fœtal heart—but we think our author has fallen into a practical error when he says there is no "placental murmur" when fibrous tumours are present. Of course we mean that a sound closely resembling the uterine sound—which our author calls placental murmur (although its mechanism is not yet clearly made out)—is undoubtedly to be heard where fibrous tumours press on the large vessels in or on the margin of the pelvis; so much alike are the murmurs in these two cases, that we have ourselves listened to the inguinal regions of two women, the one pregnant and the other with a fibrous tumour inclined to the right side, in adjoining beds, and we could not distinguish any difference between them. We look upon the so-called placental murmur as utterly useless in the diagnosis of pregnancy from other abdominal tumours.

*The Treatment of Uterine Tumours.*—Some authors suppose that fibrous tumours may be entirely absorbed—which we conclude is now to be interpreted into the fact that, under treatment, indurations of the womb have been reduced, which have been mistaken for fibrous growths.

"Local depletion, by the aid of leeches, is the best method of treatment; but these must be applied to the tumour *itself;* in a robust patient, of bloated habit, with great pain in the tumour, and with the signs of local congestion, as piles, the application of six leeches twice a-week to the neck or body of the womb will not be at all too much; but if the patient is anemic, one application weekly is sufficient. The introduction of the leeches to the tumour itself is of great importance: I have seen relays of them applied to the perinæum, rectum, and groins be of little use, while one depletion from the tumour itself has been of the utmost service. A hip bath, after the leeches come away, is very beneficial; it encourages the bleeding and relaxes the parts, and by these means removes the excessive pain which is usually present.

"During the intervals of the leechings, mercury or iodine should be applied to the womb itself, either in its pure state, or made more consistent with wax. The ointment used at the Red Lion Hospital for women is mixed with one part of the Ung. Hyd. Fort., one part of Cera Flava, and one part of lard. This is rolled up in the form of a ball, and introduced into the vagina every night, as high up as possible, in order that it may envelop the os and cervix of the uterus: this remains for twenty-four hours, when it has generally disappeared—it may then be repeated." P. 25.

Under this plan of treatment the cervix, which before may have been obliterated, again bulges below the tumour, and the mass itself sometimes rises above the brim of the pelvis, affording marked relief to the pelvic organs. It appears that Dr. Rigby has, in some cases, injected the strong mercurial ointment into the cavity of the uterus with manifest advantage. In one case, a single large solid growth appeared to separate into distinct parts and to become less. Mr. Lee discards the use of mercury internally, but he speaks favourably of the treatment by iodine, which has been of late years the most usual remedy for these growths. Mr. Lee only passingly mentions the plan of enucleating fibrous tumours when they can be got at with the scalpel, although we think he would have done wisely and contributed some useful information had he carefully investigated the cases in which this practice has been adopted.

The several varieties of polypi, as described by different authors, are succinctly noticed by Mr. Lee. Thus we have the fibrous tumour growing inwards towards the cavity of the uterus, acquiring a stalk, and thus becoming a polypus. Mr. Lee, unnecessarily we think, calls this variety polypoid; then we have the vesicular polypi, polypi from the enlargement of the Nabothian glands, fibro-cellular polypi, cellulo-vascular polypi, mucous polypi, and the channelled polypi of the cervix. This latter variety consists of a polypus springing from the neck of the womb, the substance of which is traversed by large channels, terminating by open mouths on the surface of the growth, and filled with thick clear mucus. It was first noticed and described by Dr. Oldham.

Mr. Lee has omitted to consider the influence of pregnancy on the growth of polypi, beyond, at least, a mere incidental allusion to it. The subject is interesting and important, and undoubtedly it came within the scope of the subject of the Essay.

Cauliflower excrescence of the os uteri is supposed by Mr. Lee to be a distinct affection from fungoid disease of this part, although the only distinguishing marks between the two are stated in the following paragraph.

> "If a proper examination be made on this disease, it is not difficult to distinguish it; but it has been mistaken for other growths. The most likely one is that arising from fungoid cancer: a case of this kind occurred to myself. A patient presented herself with all the symptoms of cauliflower excrescence, profuse watery discharge, no pain, health good, &c., and had from the os uteri fungoid granulations, some of which broke down upon examination: but on viewing the growths by the speculum the error was immediately corrected—they were large, flat, and not prominent." P. 93.

For our own part we can see no reason why these two affections should not be grouped together. The difference between them is very slight; and, indeed, in reading Dr. Clarke's description of the cauliflower excrescence, and comparing it with cases of fungoid disease, which are by no means rare, no essential distinction can be made between them. Perhaps the one may feel a little more like a cauliflower than the other—but the seat, development, symptoms, and general character of the growths are so much alike that it appears to us to be unnecessary to separate them. Mr. Lee has altogether omitted the description of fungoid cancer, for assuredly the encephaloid tumour, the most frequent, he says, of polypoid growths, is perfectly distinct from it. We think he might just as well have begun a chapter on "fungoid disease of the uterus," and then have added *vide* cauliflower excrescence of the os uteri. There appears to us to be a want, in this part of the Essay, of clear, well-defined views on the subject of malignant growths. If, as our author says, cauliflower excrescence is a very rare disease, and that the encephaloid growth, such as he has described it, is the next variety, we are well persuaded that he has perfectly missed a class of cases which frequently come under observation, and which the term fungoid very well designates. These growths are not confined to the os or cervix uteri, for they sometimes spring from the cavity of the womb. They occur in women with full capillary circulation, and generally wearing the aspect of good health. They are bleeding growths—bleeding when touched during a vaginal examination, or sexual coitus, or by the pipe of an injecting syringe, bleeding, too, sometimes spontaneously; and so full are they of blood-vessels that when, by the act of coughing, sneezing, or lifting weights, &c., an increased flow of blood is derived towards them, the capillaries give way, and again they bleed. They are growths, too, attended by a watery discharge, sometimes very fætid and acrimonious. They are a species of soft cancer, and are shaped and contracted by the vagina. By degrees the loss which is sustained by bleeding and discharge brings on anæmia, then the severe pains of cancer are felt, and death takes place. If a ligature is placed around even a large growth, it shrinks to nothing, and after death it appears flocculent like the villi of the chorion. Wherein is the difference between cauliflower excrescence and this fungoid disease? and yet, we repeat, these latter cases are very far from rare. The excision of these growths, or their removal by ligature, is advocated by Mr. Lee in accordance with the published cases of several obstetric physicians. Here and there an apparently complete success has followed this practice, and appears to us quite to justify the operation. Even the partial success which attends a removal of the mass prolongs life by taking away for a time the source of hæmorrhage and watery discharge. The difficulty in the operation consists in noosing or excising the entire diseased portion, and the principal danger is from hæmorrhage, which can, however, be controlled by the plug.

The second part of the work is devoted to tumours connected with the appendages of the womb, and of these the most important is the encysted ovarian dropsy. Mr. Lee has collected one hundred and forty cases of this disease, some of which have passed under his own notice, and others have been recorded in medical journals, and these have formed the data of his observations on the subject. These 140 cases are tabulated in several ways, so as numerically to prove, where the details of them have been sufficiently explicit, the various facts which are stated. Thus it is that, at the commencement, we meet with a disputed point, which is settled by a statistical table of 136 cases. Dr. Burns thinks that married women are most liable to this disease, whilst Dr. Ashwell believes that it attacks the single most frequently. Mr. Lee's table shews that, of one hundred and thirty-six cases, eighty-eight were married, eleven were widows, and only thirty-seven were single, thus giving a decided preponderance to the married. Then again, with reference to the age of patients with the disease, it appears that of one hundred and twenty six cases, only three occurred in women under twenty years of age; thirty-seven between 20 and 30; forty-five between 30 and 40; twenty-six between 40 and 50; nineteen between 50 and 60; three between 60 and 70; two between 70 and 80. Hence it seems that the disease is particularly rife during the child-bearing period; when the sexual organs are in their fullest vigour. There is another table shewing the duration of ovarian dropsy, from whence it appears "that out of 131 cases, the disease lasted only one year in 38, only two years in 25 : 17 patients survived three years, 10 four years, 3 five years, 5 six years, 4 seven years, 3 eight years, 1 nine years, 1 ten years, 1 eleven years, 5 twelve years, 5 sixteen years, 1 twenty years, 1 twenty-two years, 2 twenty-five years, and 1 thirty years."

Table No. 4, is headed "of the imputed causes of ovarian dropsy in thirty-six cases." Fourteen cases are ascribed to marriage or its consequences—that is, five followed marriage, and the patients regarded it as the cause, and nine followed parturition. Seven cases were supposed to be caused by a sudden suppression of the menses. Two cases were traced to abortion; three to exposure to cold; two to falls or blows; one to a violent fit of anger; one to an eruption, and one to disappointed love. There were only two which were assigned to the catamenial decline, which Denman thought the most frequent cause. A Table is prepared to shew the frequency of ovarian disease in the right, left, or both ovaries, the inference from which is, that the right is affected in frequency to the left, and to both ovaries as 50 is to 35 & 8. This conclusion is at variance with Mr, B. Cooper's results in 50 cases, from which it appeared that, the left ovary was more commonly diseased than the right. We look upon it that Mr. Lee's table is no more secure with his 93 cases of a truthful result than Mr. Cooper's, and that the question is still quite undecided. We shall not follow our author into the pathology of ovarian dropsy, which has been so fully written upon by several authors, more especially by Dr. Hodgkin. The diagnosis of this disease is carefully described; but the most important part of this Chapter is on its treatment. Mr. Lee has not entered so fully into the subject of the treatment of this disease in its early stages as he should have done. A little of the industry which has been spent on the attractive subject of Ovariotomy, might have been advantageously employed in collecting some facts on the influence of leeching, mercury, and counter-irritants, in checking the growth of small tumours. It is well known that the late Dr. Hamilton treated ovarian cysts by bandaging, percussion, and the internal use of the muriate of lime; but the success which followed its adoption in Dr. Hamilton's hands has not been copied in England, and it has fallen into desuetude. Mr. J. Brown of London is said to have cured several cases by tapping the cyst, after giving the patient mercury and diuretics, and then tightly bandaging the abdomen. We transcribe a short outline of his practice from Mr. Lee's book.

"I divide," says Mr. Brown, "my treatment into constitutional and local treatment, and treatment after tapping.

"1. The constitutional one consists in the administration of mercurials, internally as alteratives, and externally by friction over the abdomen, and continued until the gums are slightly but decidedly affected: and this must be continued for some weeks. I lay particular stress upon this point: at the same time diuretics must be given, and after the first week tonics must be combined with them. The food should consist of light animal diet, and should be unstimulating; and the patient should take daily exercise in the air."

"2. Local treatment. This consists of the careful application of a tight flannel bandage, so as to produce considerable pressure over the tumour. When it is found that the abdominal action has been checked by a positive decrease in the tumour, and a continuation of such decrease, or by a positive non-increase for some weeks, then the cyst should be tapped and all its fluid evacuated."

"3. Treatment after tapping consists of accurate padding and tight bandaging over the cyst and body generally for two or three weeks; and the medicines and position ought to be continued for at least six weeks. I would particularly wish to enforce the importance of the after-treatment, as on that depends very much the success or failure of the case." P. 161.

Certainly, the system of tight bandaging and padding, after a patient has been salivated is very formidable, and the recital of the sufferings and danger to which patients have been exposed is sufficient to make any judicious practitioner seriously pause before he sanctions it. Mr. Lee seems to question the successful issue of Mr. Brown's cases, and on the authority of a physician, whose name however does not appear, he mentions two of the "successful" cases—one of which has again been tapped; and, after the death of the other, the cyst was found as large as ever. Then, again, much doubt seems to hang on the genuineness of the cases, as to whether they have all been ovarian cysts, or some other abdominal enlargement, so that altogether, Mr. Brown's success appears to have been sometimes temporary only and altogether very equivocal.

Of the surgical means for the cure of ovarian dropsy, there are two particularly described, viz. tapping and the excision of the cyst. As a palliative means tapping is sometimes very successful; and cases are recorded where patients have lived many years, the fluid of the cyst being frequently drawn off by the trocar. In Mr. Martineau's case, 13 hogsheads of fluid were taken away from an ovarian cyst in eighty different tappings. But tapping is not without its danger—syncope, and speedy exhaustion may sometimes follow the evacuation of the fluid—a large vessel may be punctured, the cyst may inflame, and peritonitis may ensue. It sometimes is only partially successful, when the tumour is multilocular. Mr. Lee is favourable to an early recourse to paracentesis; and he thinks that, when the tumour can be felt from the posterior wall of the vagina, and fluctuation is distinct, it ought to be tapped. We could have wished Mr. Lee to have collected more facts, to shew the results of this early tapping, on the value of which he speaks authoritatively and without doubt.

In order to clear the way for a correct appreciation of the operation of ovariotomy, Mr. Lee has collected a number of cases in which tapping was performed, and he has noted the duration of life after it has been had recourse to.

Putting together 57 cases of this kind, taken from Mr. Lee's own table and Mr. Southam's, in which death occurred, it appears "that 24 of the 57 died after the first tapping—that they all died in eight months—that 20 of the 24 died within one month—and 12 of the 20 within seven days." Mr. Lee thinks it possible that many of the cases from which his table has been framed, may have been published as peculiar cases, which would of course vitiate the truth of the conclusions. Many eminent men whom he spoke to about it, thought the mortality after the first tapping was too great; but he is himself disposed to think it correct. We must own that the numbers appear very startling, and do not accord with our general impression; but we think that it discloses a far larger mortality, admitting even the possible errors in it, than is generally supposed. Our author concludes that, "taken at its best, tapping is a very dangerous means of palliating ovarian dropsy; that, when it is had recourse to, it will have to be frequently repeated; that the relief afforded between each operation will become gradually less, and the dangers consequently greater. This, then, is a valuable argument in favour of some other means of treating ovarian dropsy."

We must now advert to the other operation—namely, the removal of the disease by excision. Mr. Lee's views on this subject are founded on one hundred and eighteen cases in which gastrotomy has been performed, which he has collected with much diligence, and carefully tabulated. Of these 78 have recovered, and 40 have died; in ninety-two the tumour has been removed: in nineteen it was not extracted; and in six there was no tumour to be found. But we meet at once with a source of error in the fact, that (according to Mr. Phillips, Dr. Bird, and Mr. Lee) there are several unsuccessful operations which are not recorded—augmenting, of course, the mortality of the operation, without, we fear, being paired by a corresponding omission of the more fortunate cases. Of these 118 cases of gastrotomy, sixty-nine were for the removal of encysted tumours of the ovary; sixteen for solid tumours of the ovary; six for uterine tumours (fibrous growths); one for an omental tumour; one for the cyst of an ovarian abscess; in six no tumour was found; and the particular disease is not mentioned in 19. Of the 69 cases of encysted dropsy, 48 recovered and 21 died. Of the 16 cases of solid tumours, nine recovered and seven died; and of the six cases of fibrous tumour of the uterus, two recovered and four died.

There are two or three important and obvious points on which Mr. Lee makes some observations. One of them is on the mortality after ovariotomy, another, on the difficulties in the diagnosis of ovarian dropsy, and particularly on the means of ascertaining the presence of adhesions, which have proved the principal hindrance in the uncompleted cases. Mr. Lee estimates the average mortality in ovariotomy as one in three; and, comparing this with the mortality in other capital operations, it appears to be rather under than above the mark. Malgaigne has computed the deaths after amputations, of all kinds, in the Parisian hospitals, as four in every ten cases; and it is much the same in the Glasgow and Edinburgh Infirmaries. In the tying of arteries, the deaths have been $3\frac{1}{2}$ in 10; and in cases of hernia, 5 in 10.

The tendency which ovarian tumours have to contract adhesions to the structures and viscera adjacent to them, is a formidable impediment to their extraction. In the cases which have been recorded, they were found to exist in more than one-half of the number. They may be numerous and firm, without any previous known inflammatory attack; and it is a mistake to suppose that tapping causes adhesion. In the latter case, the collapsed and empty cyst sinks down into the pelvis, away from the opening which has been made by the trocar.

There have been several supposed signs of adhesion, on which, however, as single signs, no absolute reliance can be placed. An ovarian sac may be moveable, and yet adhesions, probably long and firm, may be present. Dr. Bright's sign of the new-leather creaking or crepitation as demonstrating adhesions, may not be heard; first, because it is principally to be heard if the adhesions are recent, or, according to Mr. Southam, only when fluid is present; and then, again, it may be present, but so deeply placed behind the sac, that it cannot be perceived by the ear. A valuable sign of the presence of adhesions in front was communicated to Mr. Lee by Dr. Bird.

"When an ovarian sac has attained a size which is productive of great inconvenience and distress to the neighbouring organs, the parietes of the abdomen become greatly attenuated, and the space between the two recti abdominalis is much enlarged: this is well seen if the patient be told, while lying on her back, to raise herself into the sitting posture without the assistance of her arms; and if the sac within be free in its motions it will immediately be protruded through the space between the two recti muscles, and produce an oval enlargement; but supposing the cyst to be intimately adherent in front, no such bulging will take place." P. 189.

The action of the diaphragm on the tumour will help to determine the existence of adhesions.

"Another symptom of this sort is valuable, and that is the action of the diaphragm upon the tumour. If the measurement of the abdomen be obtained after the patient has taken a deep inspiration, and again after a full expiration, you will find, when the cyst is free, that the two measurements frequently vary an inch, sometimes more; shewing that the diaphragm in the inspiratory movement had driven down the unattaehed cyst, while it being free, the expiratory effort allowed it to repossess its original position in the abdomen." P. 189.

Tapping the cyst is a practical means of learning the presence of adhesions.

"On the withdrawal of the fluid, the walls of the abdomen are observed to follow closely the contracting cyst, when adhesions are present, and have externally a drawn-in and puckered appearance, while the cyst does not descend into the pelvis; whereas, when the cyst is free from adhesions, it may be found after its evacuation low in the pelvis, forming a hard tumour at the lower part of the abdomen, while the walls of the abdomen may remain free." P. 190.

It is, however, in a careful collection of several of these signs that the complication is to be made out.

"But, although the dependence on these symptoms singly may lead us into error, the combination of many of them will generally be conclusive, supposing the patient, when rising by her own exertions, protrudes the cyst as an oval bulging tumour through the space left by the separation of the recti. That on a deep inspiration the tumour is pressed downwards more into the cavity of the abdomen, and then recedes on an expiration; that the bladder is free, and can ascend into the anterior part of the abdomen when filled with air; that all crepitation is absent, and the tumour tolerably moveable: then we may with satisfaction say that adhesions do not exist. Another additional evidence would be, if the patient had been previously tapped, and the sac had entirely disappeared after the operation." P. 194.

Leaving the question as to whether ovariotomy is expedient or not until the conclusion of the chapter, our author describes the two different modes of operating, which are respectively called the major and minor operations. When the incision has exceeded six inches in length Mr. Lee has classed the case as belonging to the former, whilst an incision under six inches in length is ranked as a minor operation. Eighty-five cases have been operated on by the large opening, and the mortality has been one in three.

Twenty-three cases have been relieved by the minor operation, and the deaths have been one in six. It is still a disputed point which of the two is the most feasible operation. Dr. Clay, Mr. Walne, and others preferring the major, Mr. Jefferson, Dr. F. Bird, and others holding to the minor. Mr. Lee argues for the latter, and the obvious fact that the latter may, in case of need, be converted into the former is a strong general argument in its favour.

Mr. Lee has learned, by private communications from Mr.' Lane, Dr. Clay and Dr. Bird, that the women on whom they operated successfully continue to enjoy good health. We conceive this to be a most important addition, and we think that these and other operators should concisely publish the after history of their cases.

Mr. Lee thinks that, in the majority of cases, " ovariotomy is most decidedly unjustifiable," and he arrives at this conclusion from the difficulty of the diagnosis in the disease, and from the frequent existence of adhesions. He thinks the increased mortality, when adhesions have been present, disqualifies such cases for this means of relief, and when the diagnosis is obscure he would of course avoid so frightful an operation. He talks in very contradictory terms of the value of the uterine sound in clearing the diagnosis in these cases, for in the text he says, that " no tumour of the uterus ought to be mistaken for ovarian disease since the introduction of the uterine sound by Professor Simpson;" and then, in a foot-note, it appears that " he and several others, men in the constant habit of using the sound, were deceived in a case of ovarian dropsy." We think he has overstrained the value which this really useful obstetric instrument affords in these cases.

Mr. Lee, however, is " decidedly of opinion that in some cases the operation is *very justifiable.*" It is particularly so where the " cyst is single and uncomplicated with hard matter, and the powers of life active."

The treatment which Dr. F. Bird adopts after extirpation consists principally in being able pretty quickly so to raise the temperature of the room as to cause profuse sweating, for which purpose also he gives the patient plentifully of ice. Should febrile symptoms arise, he brings on the free action of the skin, and watches his patient unremittingly for some days.

We have thus brought before our readers the principal results which flow from Mr. Lee's compilation on this interesting subject, and with it our observations on his Dissertation. We congratulate him on the honourable prize which he has won, and we cordially wish him success in the department of medicine which he appears to have chosen. But we seriously advise him, while he yet has time, to cultivate a knowledge of general literature, not only for the alluring purposes of disciplining, storing and enlarging the mind, but for the lower and indispensable object of writing correctly.

